# Supplementary material for: Contrasting inequality in human exposure to greenspace between cities of Global North and Global South
Source: Nat Commun. 2022 Aug 8;13:4636. doi: 10.1038/s41467-022-32258-4 (PMC9360024; doi:10.1038/s41467-022-32258-4)
Supplement: Supplementary file 1 — Supplementary Information [file 41467_2022_32258_MOESM1_ESM.docx]

Supplementary Information for

**Contrasting inequality in human exposure to greenspace between cities of Global North and Global South**

Bin Chen^1,2,3,4,*^, Shengbiao Wu^1^, Yimeng Song^5,6^, Chris Webster^2,4,7^, Bing Xu^8^, Peng Gong^2,3,9,*^

1. Future Urbanity & Sustainable Environment (FUSE) Lab, Division of Landscape Architecture, Department of Architecture, Faculty of Architecture, The University of Hong Kong, Hong Kong SAR

2. Urban System Institute, The University of Hong Kong, Hong Kong SAR

3. Institute for Climate and Carbon Neutrality, The University of Hong Kong, Hong Kong SAR

4. Institute of Data Science, The University of Hong Kong, Hong Kong SAR

5. Department of Land Surveying and Geo-Informatics, The Hong Kong Polytechnic University, Hong Kong SAR

6. School of the Environment, Yale University, New Haven, CT 06511, USA

7. HKUrbanLabs, Faculty of Architecture, The University of Hong Kong, Hong Kong SAR

8. Department of Earth System Science, Ministry of Education Key Laboratory for Earth System Modeling, and Institute for Global Change Studies, Tsinghua University, Beijing 100084, China

9. Department of Geography, and Department of Earth Sciences, The University of Hong Kong, Hong Kong SAR

*Corresponding authors: Bin Chen ([binley.chen@hku.hk](mailto:binley.chen@hku.hk)) and Peng Gong ([penggong@hku.hk](mailto:penggong@hku.hk))

**1. Supplementary Information**

**1.1. Calculation of Gini Index**

As shown in Fig. S14, Gini index is mathematically calculated as the ratio of the area that lies between the line of equality and the Lorenz curve (region *A*) over the total area under the line of equality (region *A* plus region *B*):

$Gini=\frac{Area_{A}}{Area_{A}+Area_{B}}$ (S1)

where ${Area}_{A}$ and ${Area}_{B}$ represent the areas of regions *A* and *B*, respectively.

Since the scale of both x- and y-axis in Supplementary Fig. 5 ranges from 0 to 1, we have *Area_A_* + *Area_B_* = 0.5. Thus, the Gini index can be formulated as:

$Gini=\frac{Area_{A}}{0.5}=\frac{0.5-Area_{B}}{0.5}=1-2\times Area_{B}$ (S2)

We moved to the area calculation of region *B* that is bounded by the cumulative share of greenspace exposure and the cumulative share of residents from lowest to highest greenspace exposure. To this end, we first used the numerical integration approach to calculate the area of trapezoid *B_i_* that contributed by the *i*th resident, and then summed each trapezoid area across all residents. The area of trapezoid *B_i_* (*Area_Bi_*) is calculated as follows:

$Area_{Bi}=\frac{1}{2}\times\left(\frac{\sum_{j=1}^{i-1} g_{j}}{\sum_{j=1}^{n} g_{j}}+\frac{\sum_{j=1}^{i} g_{j}}{\sum_{j=1}^{n} g_{j}}\right)\times\frac{1}{n}$ (S3)

where $g_{j}$ is the greenspace that exposed to *j*th resident and *n* is the total resident number.

Thus, the area of region *B* is calculated as

$Area_{B}=\sum_{i=1}^{n} Area_{Bi}=\sum_{i=1}^{n} \frac{1}{2}\times\left(\frac{\sum_{j=1}^{i-1} g_{j}}{\sum_{j=1}^{n} g_{j}}+\frac{\sum_{j=1}^{i} g_{j}}{\sum_{j=1}^{n} g_{j}}\right)\times\frac{1}{n}$ (S4)

By substituting Eq. (S4) into Eq. (S1), we finally derive the Gini index using the following formula.

$Gini=1-\frac{\sum_{i=1}^{n} \sum_{j=1}^{i-1} g_{j}+\sum_{i=1}^{n} \sum_{j=1}^{i} g_{j}}{n\times\sum_{j=1}^{n} g_{j}}$ (S5)

**1.2. Calculation of spatially explicit greenspace exposure**

The greenspace exposure assessment through Eq. (2) is conducted at the aggregation level, for example, at country, state, county, and city scales. In order to derive the spatially explicit map of greenspace exposure, we used the normalized greenspace exposure to calculate the pixel-based greenspace exposure through Eq. (S6),

$GE_{i}=\frac{Pop_{i}}{\overline{Pop}}\times GC_{i}$ (S6)

where *GE_i_* represents the greenspace exposure at the *i*th pixel, *Pop_i_* represents the total population of the *i*th pixel, *GC_i_* represents the greenspace coverage at the *i*th pixel, and $\bar{Pop}$ represents the pixel-based mean population in the corresponding administrative unit.

In this way, we can generate the comparison between greenspace coverage and greenspace exposure over different scales (Supplementary Figs. 5-8). It should be noticed that by averaging the pixel-based normalized greenspace exposure, we can also achieve the overall greenspace exposure assessment at the city level.

**1.3. Comparison between tree and shrub/grass exposure**

We first categorized two key components of urban greenspace into 1) forest/tree (shadowed greens), and 2) shrub/grass (non-shadowed greens) from WorldCover maps and calculated the different greenspace exposure using the Eq. (2). We further calculated the percentage of tree exposure regarding the total greenspace exposure for global 1028 cities and quantified the correlation between these two types of greenspace exposure and the total greenspace exposure.

**1.4. Sensitivity of buffer distance and grid size to the assessment of greenspace exposure inequality**

We conducted a set of sensitivity experiments to investigate the impact of buffer distance and grid size on the statistical assessment of greenspace exposure inequality. Specifically, as for buffer distance, we increased the parameter of buffer zones from 100 m to 2000 m with an interval of 100 m and calculated the corresponding Gini values of greenspace exposure. As for grid size, we increased the parameter from 100 m, 250 m to 2000 m with an interval of 250 m and calculated the corresponding Gini of greenspace exposure. The results of variation of Gini of greenspace exposure to buffer distance and grid size are presented in Supplementary Fig. 21.

**Supplementary figures**


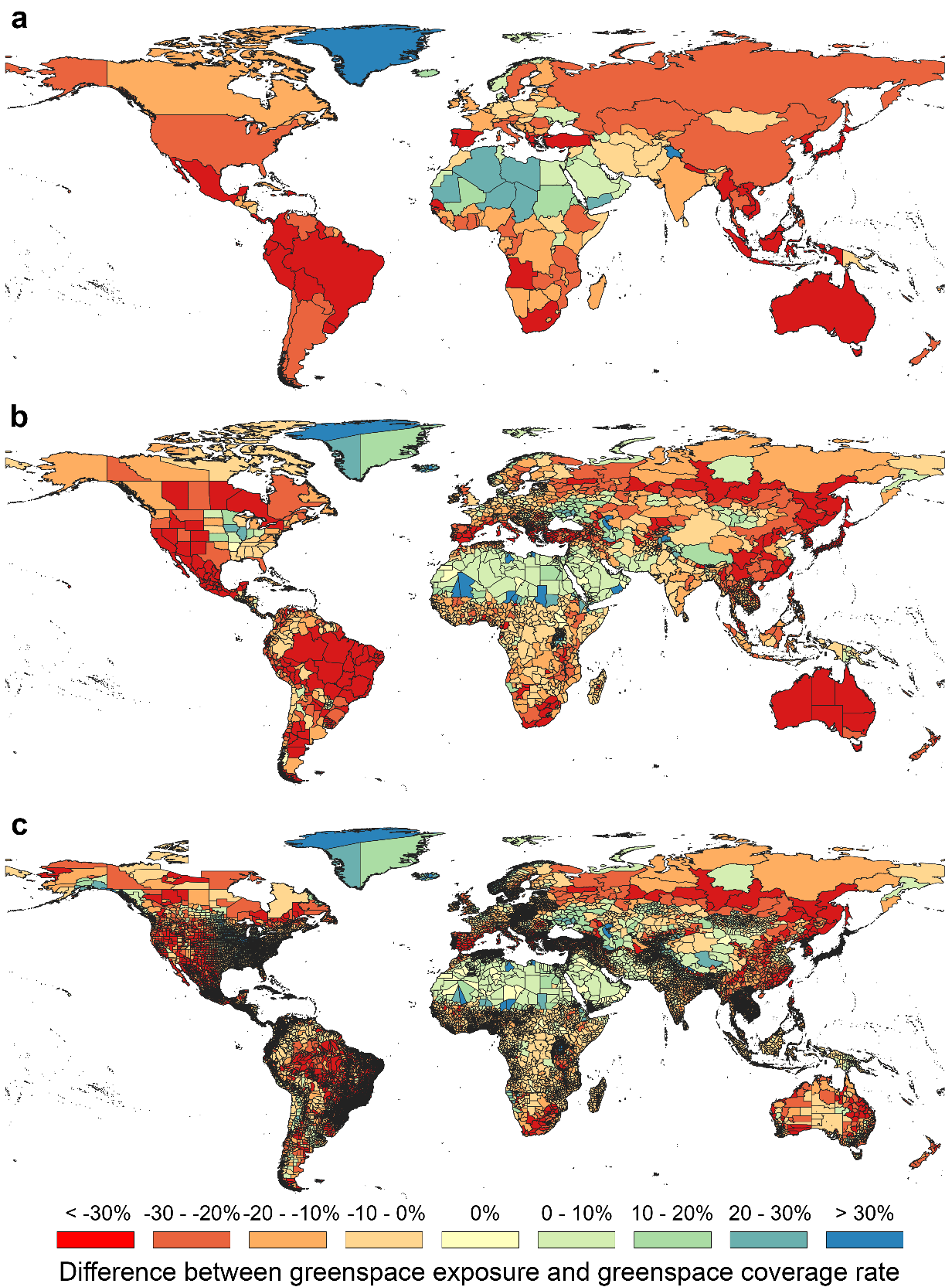


**Supplementary Fig. 1. Difference between population-weighted greenspace exposure and greenspace coverage rate across different administrative divisions of (a) country, (b) state, and (c) county.**

**
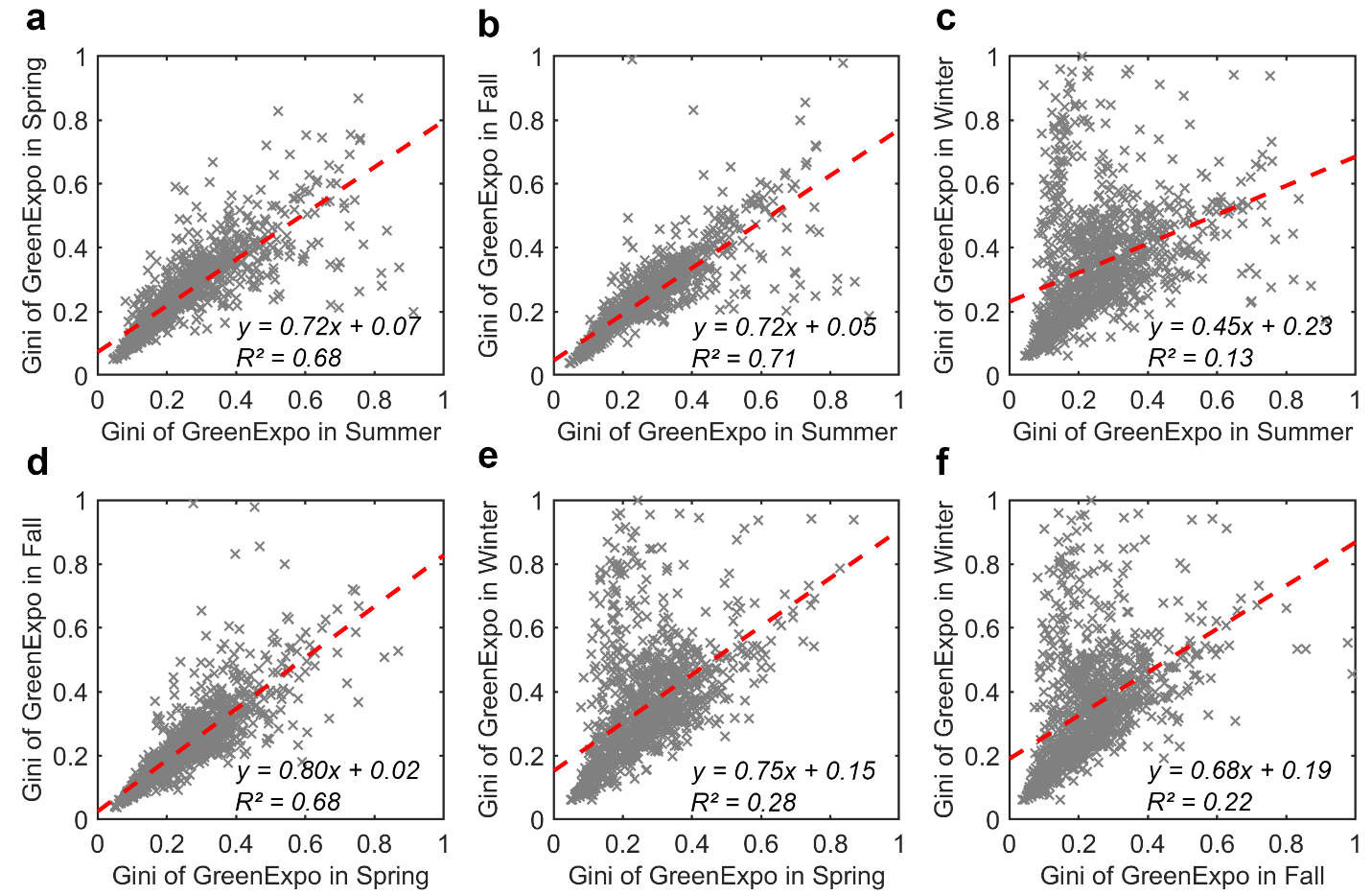
**

**Supplementary Fig. 2. Comparison of greenspace exposure inequality measured by Gini index between different seasons for global urban areas. a,** Summer vs Spring. **b,** Summer vs Fall. **c,** Summer vs Winter. **d,** Spring vs Fall. **e,** Spring vs Winter. **f,** Fall vs Winter. The fitting lines are derived from a linear regression to justify the correlation of Gini index of greenspace exposure between two seasons.


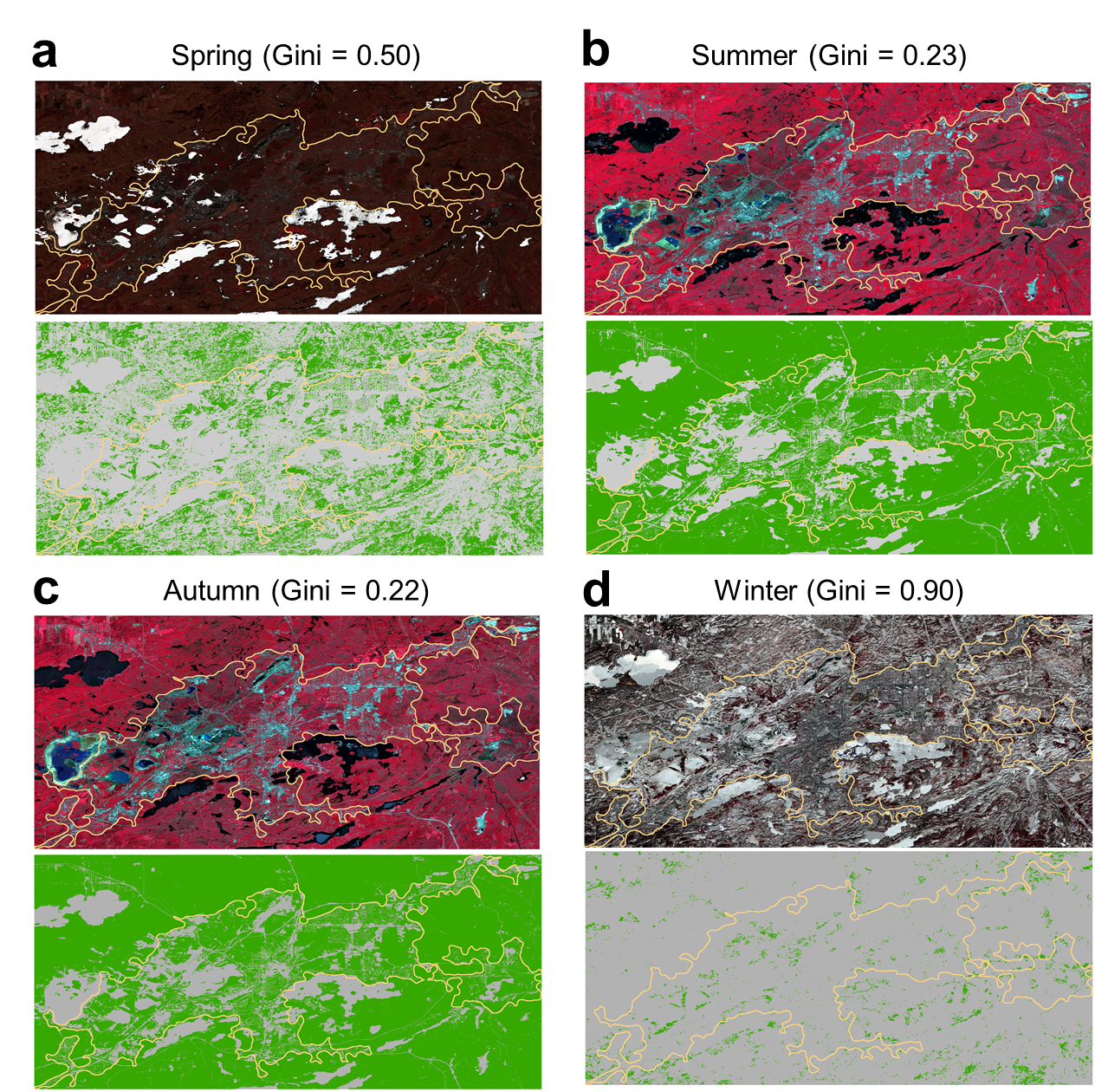


**Supplementary Fig. 3. Seasonal changes of greenspace coverage and the associated Gini index of greenspace exposure using an example in Greater Sudbury, Ontario, Canada.**

**
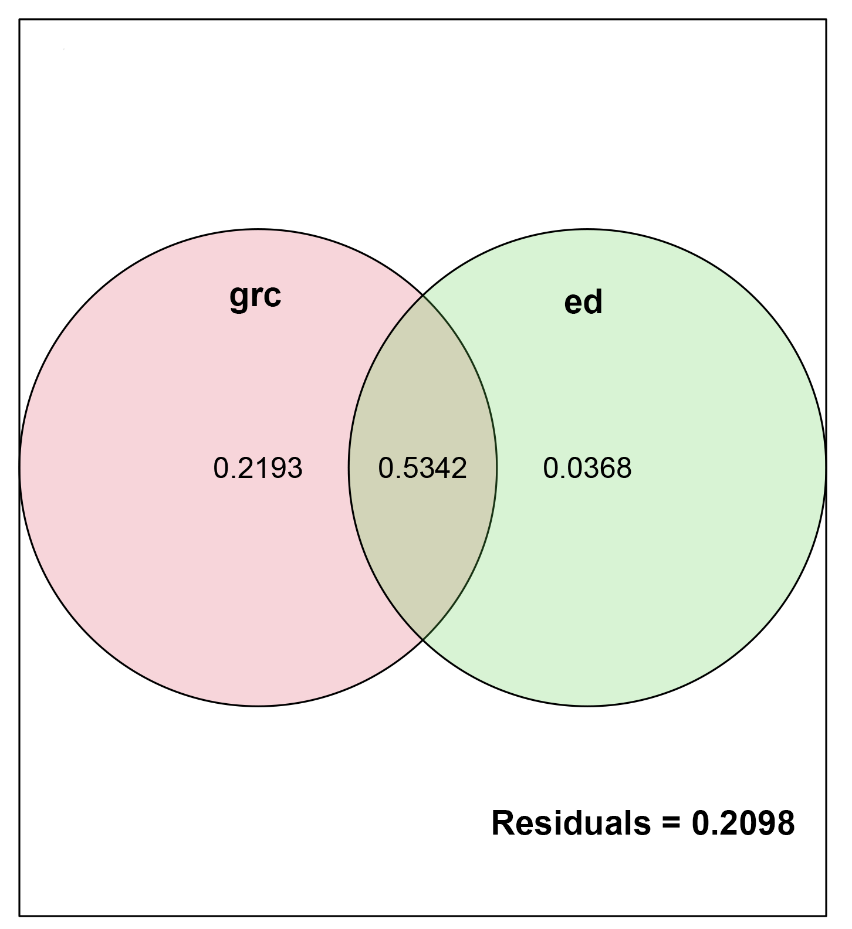
**

**Supplementary Fig. 4. Results of variable partition (100%) for explanatory variables** **of** greenspace coverage rate (gcr) and edge density (ed).


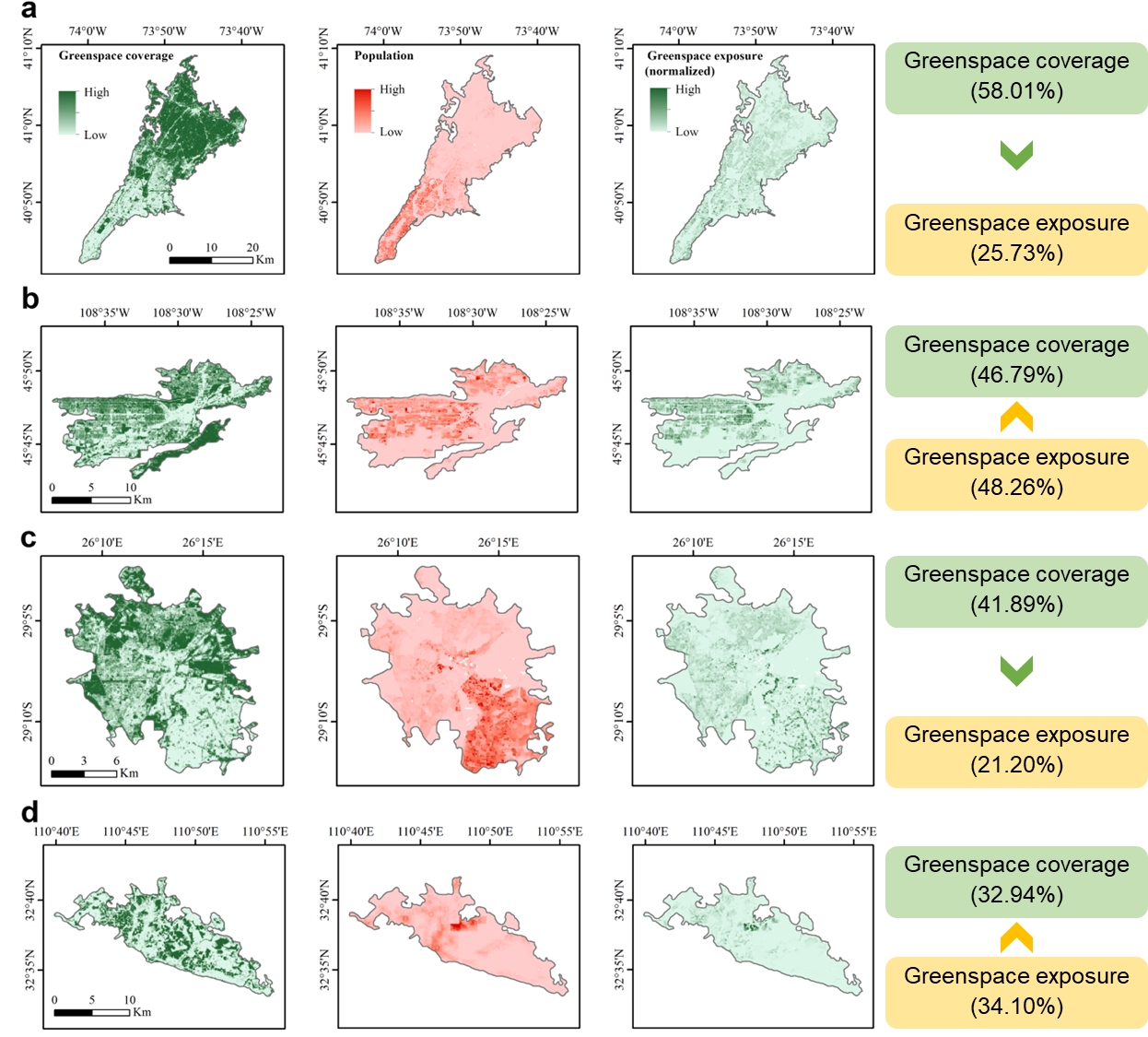


**Supplementary Fig. 5. City-level comparison of greenspace coverage and greenspace exposure in (a) New York City, New York, United States and (b) Billings, Montana, United States for the Global North, (c) Bloemfontein, Free State, South Africa and (d) Shiyan, Hubei, China for the Global South.** Panels from the left to right represent the spatial maps of greenspace coverage, population, normalized greenspace exposure. It should be clarified that greenspace exposure assessment is conducted at the city scale. In order to derive the spatially explicit map of greenspace exposure, we used the normalized greenspace exposure (population_i/mean population * greenspace coverage_i) for visual comparison. By averaging the pixel-level normalized greenspace exposure, we can achieve the overall greenspace exposure assessment at the city level**.**


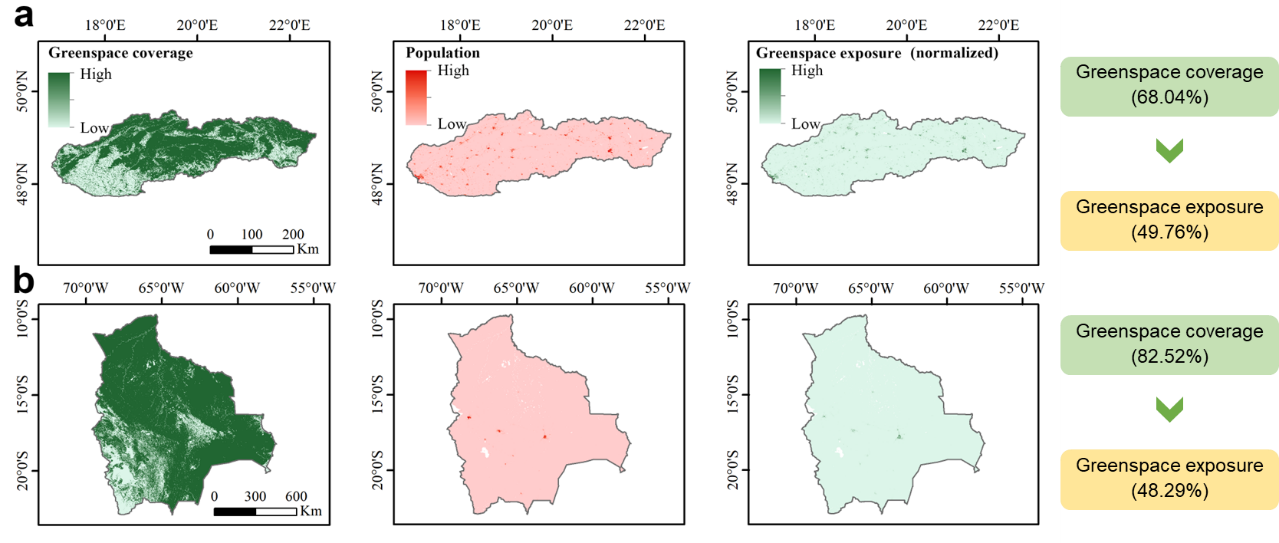


**Supplementary Fig. 6. Country-level comparison of greenspace coverage and greenspace exposure in (a) Slovakia (Global North) and (b) Bolivia (Global South).**


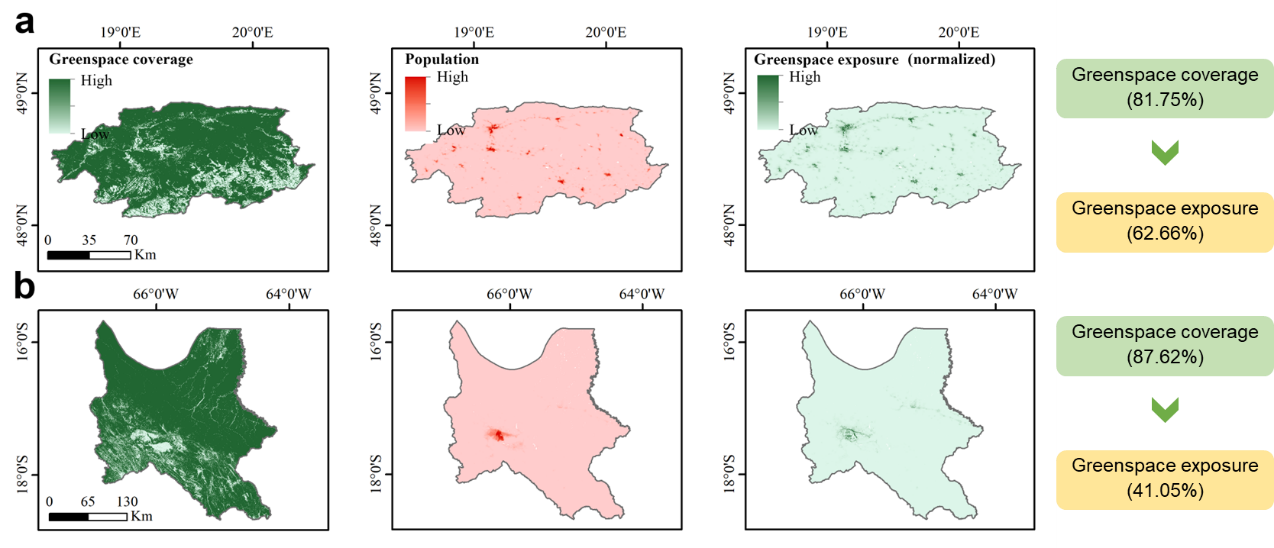


**Supplementary Fig. 7. State-level comparison of greenspace coverage and greenspace exposure in (a) Banska Bystrica, Slovakia (Global North) and (b) Cochabamba, Bolivia (Global South).**


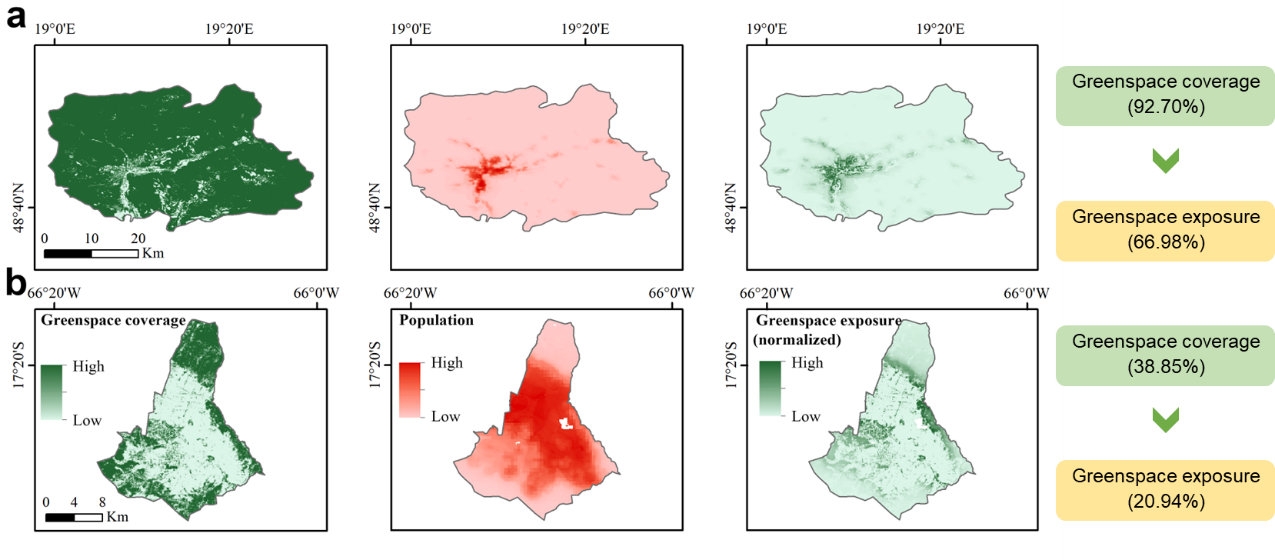


**Supplementary Fig. 8. County-level comparison of greenspace coverage and greenspace exposure in (a) Banska Bystrica, Banska Bystrica, Slovakia (Global North) and (b) Cercado, Cochabamba, Bolivia (Global South).**

**
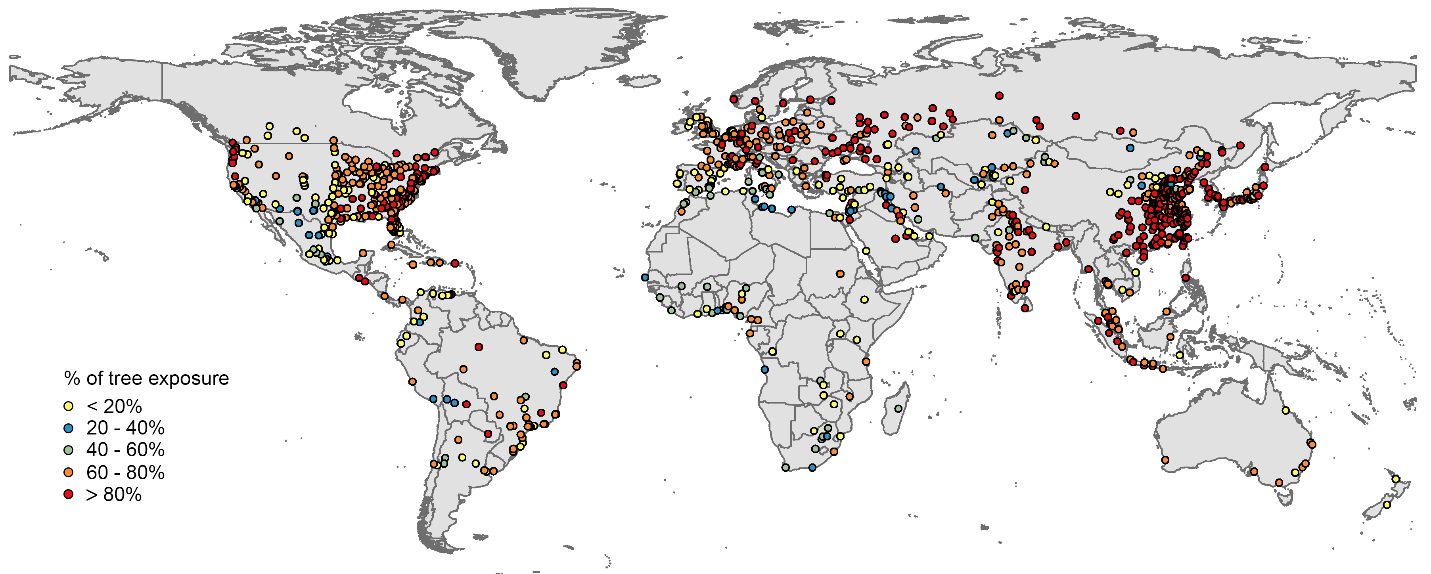
**

**Supplementary Fig. 9. Percentage of tree exposure regarding the total greenspace exposure levels for global 1028 cities.**

**
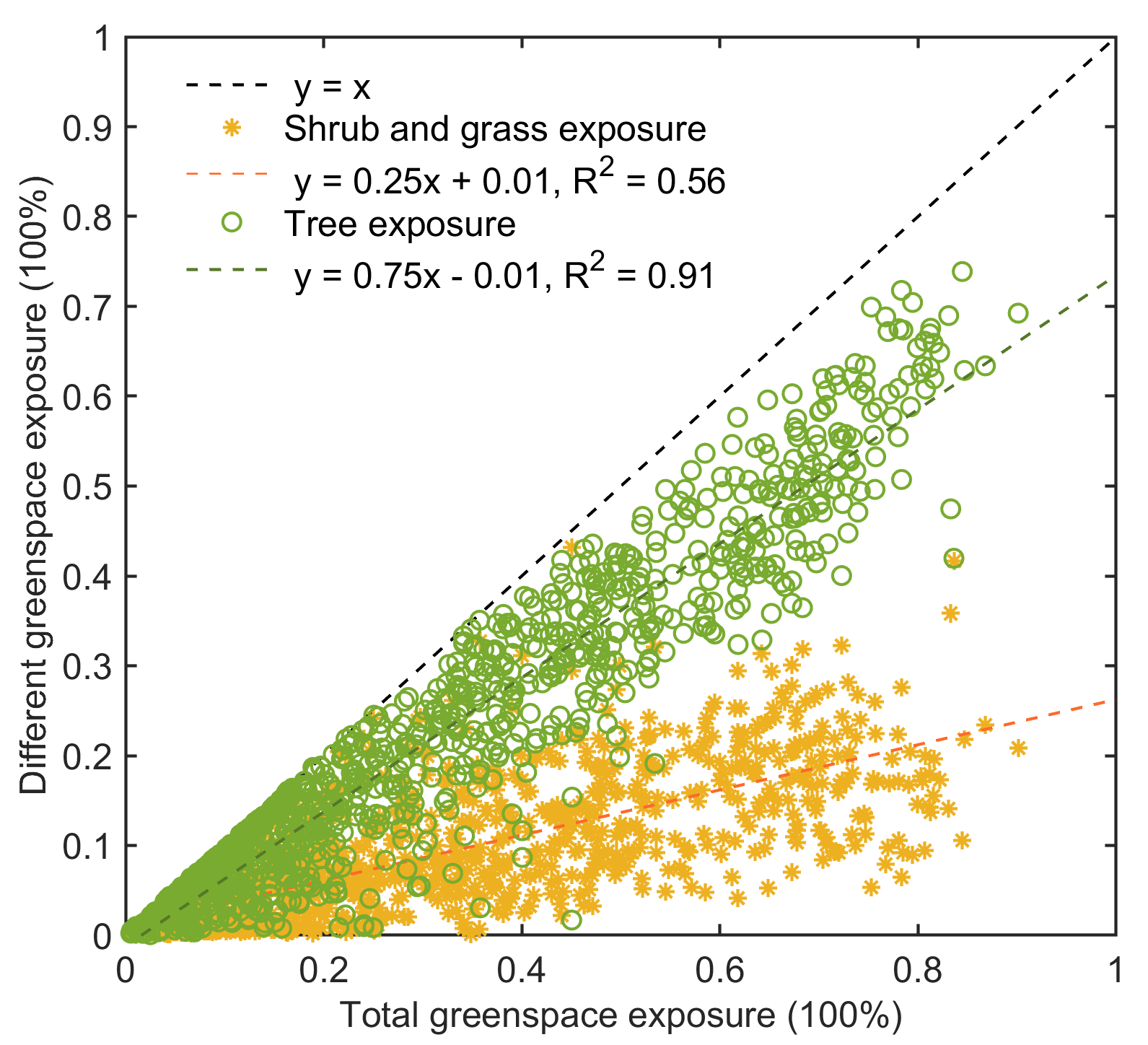
**

**Supplementary Fig. 10. The association of tree exposure (green dots) and shrub/grass exposure (yellow dots) with total greenspace exposure for global 1028 cities.**


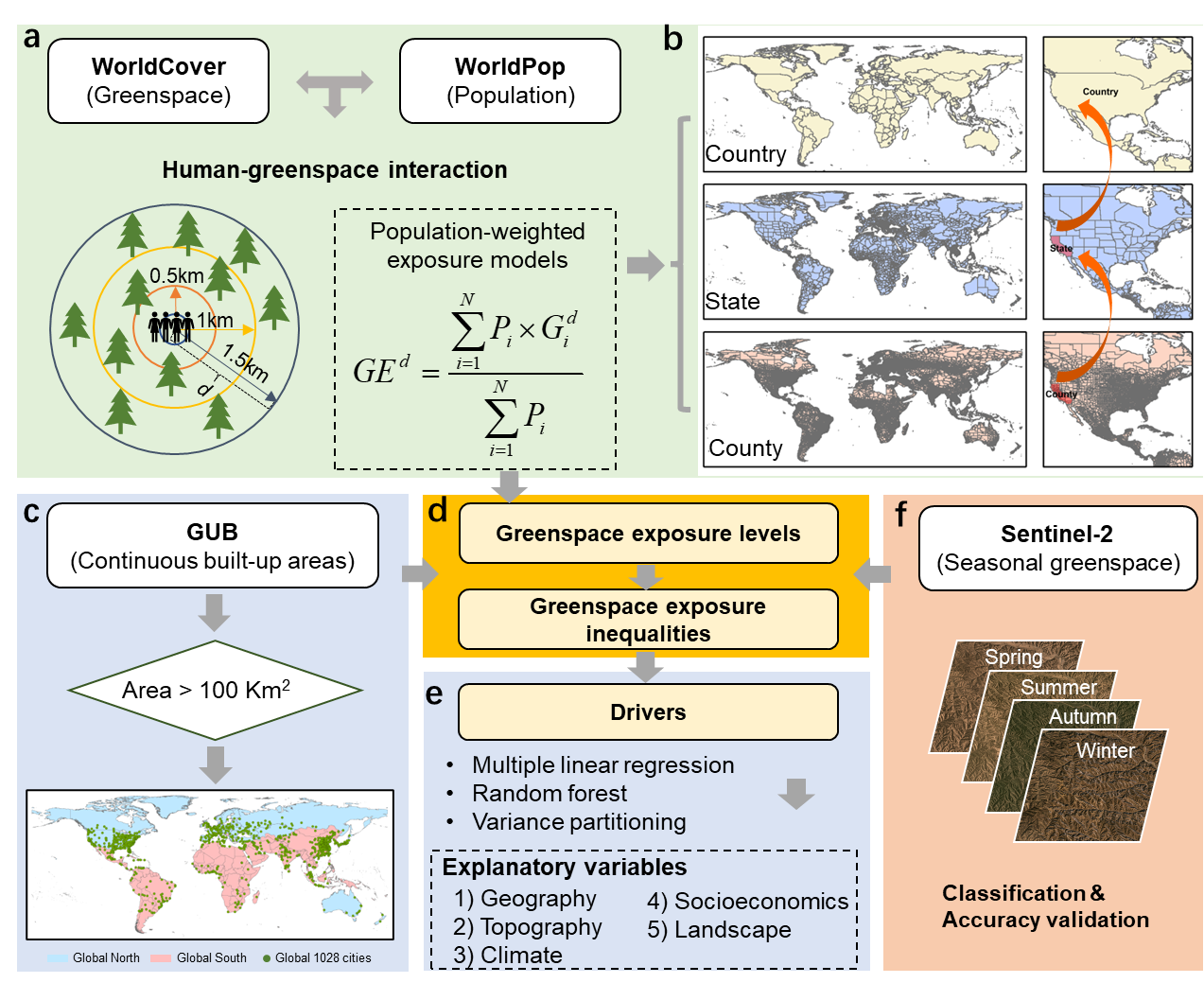


**Supplementary Fig. 11. Flowchart of the research design with six major steps.** (**a**) Modeling human-greenspace interaction with the population-weighted exposure models, (**b**) global differences in human exposure to greenspace at country, state, and county scales, (**c**) selection of global 1028 cities, (**d**) assessment of greenspace exposure levels and inequalities in global 1028 cities, (**e**) analysis of drivers for greenspace exposure inequalities, and (**f**) seasonal change in greenspace exposure levels and inequalities. Specifically, steps (**a-b**) are designed to address the 1st research question, steps (**c-e**) are designed to address the 2nd research question, and steps (**c-d**) and (**f**) are designed to address the 3rd research question.


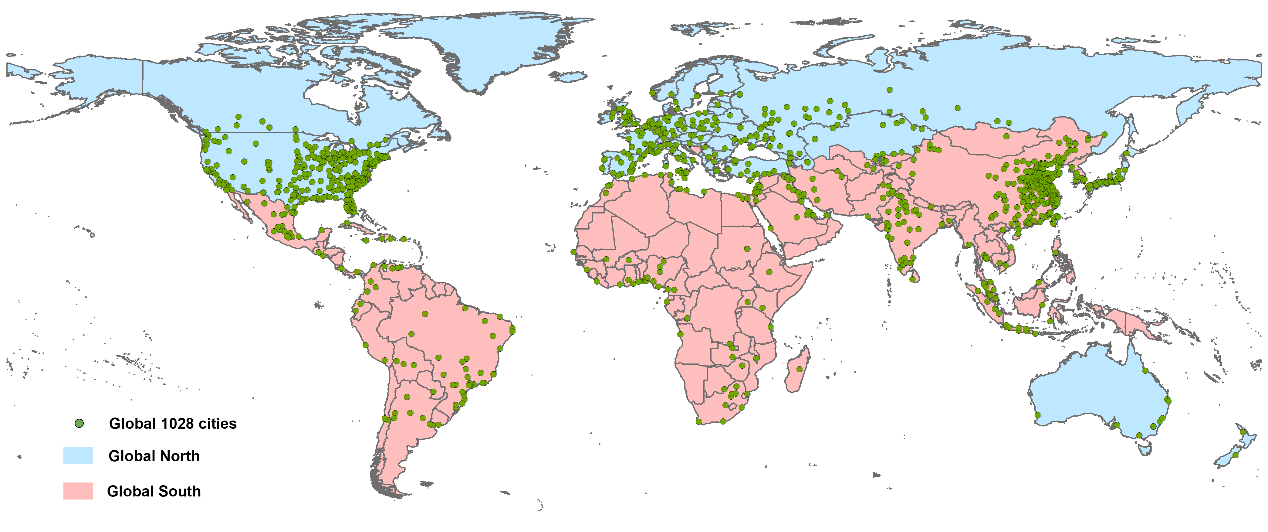


**Supplementary Fig. 12. Geographic location of global 1028 cities overlaid on the classification of Global North and Global South.**


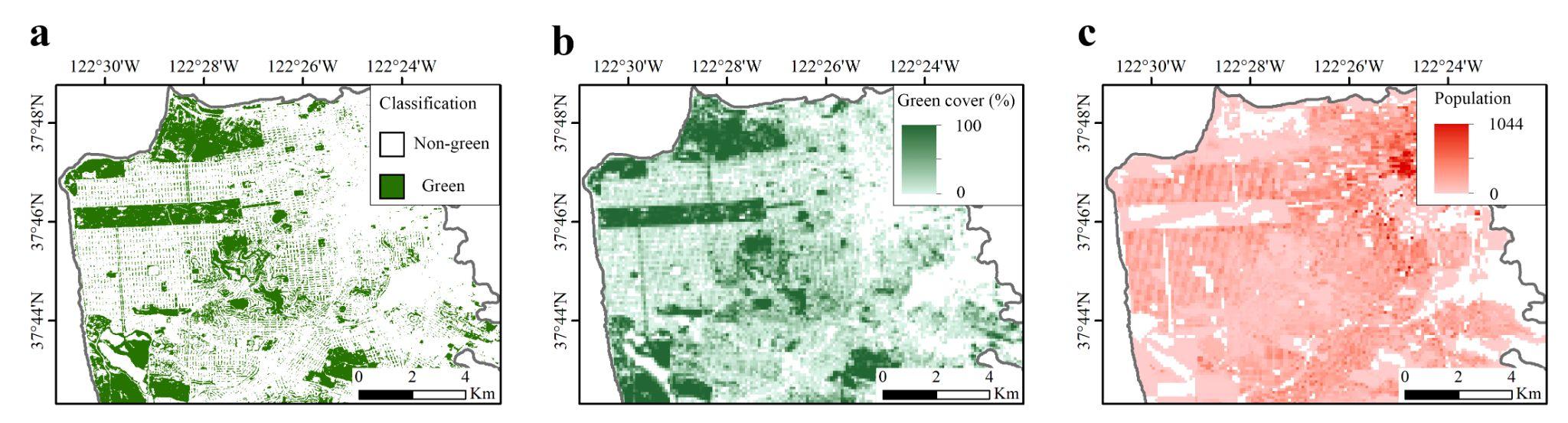


**Supplementary Fig. 13. Illustrative diagram of aggregating (a) 10-m WorldCover greenspace map to (b) 100-m fractional greenspace coverage map, to be spatially consistent with (c) 100-m WorldPop population grid data, using the City of San Francisco as an example.**


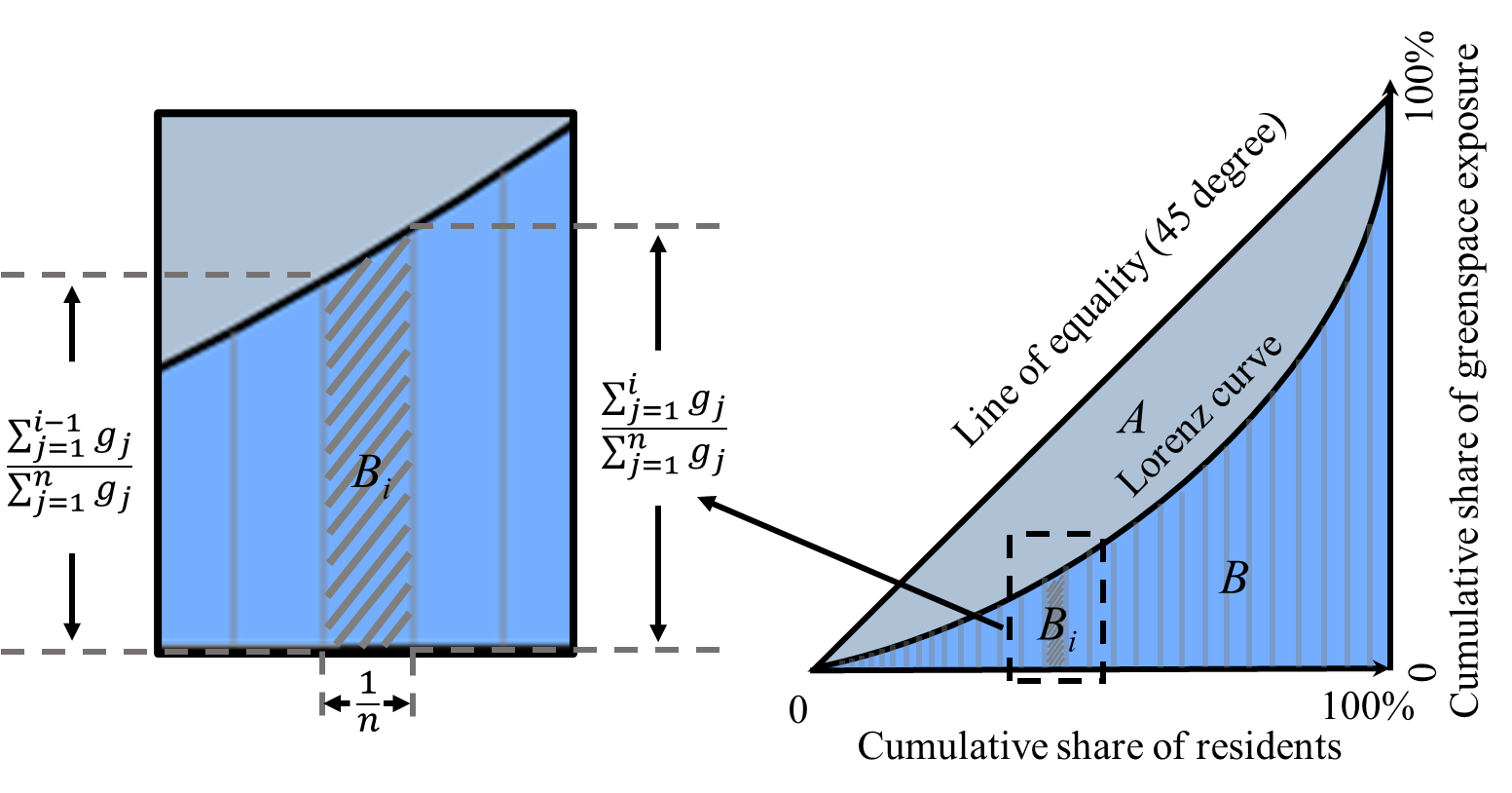


**Supplementary Fig. 14. Illustrative diagram of Gini index-based inequality assessments of greenspace exposure.** The Gini index is defined as the ratio of the area that lies between the line of equality and the Lorenz curve (region *A*) over the total area under the line of equality (region *A* plus region *B*), where Lorenz curve plots the proportion of the greenspace exposure (y-axis) that is cumulatively shared by the residents (x-axis). *B_i_* indicates the contribution of *i*th residents to the accumulated greenspace exposure and is estimated by the trapezoid area as shown in the left panel, where *g_i_* represents the greenspace that is exposed to *i*th resident, and *n* represents the total resident number. Y-axis shows the cumulative share of greenspace exposure; X-axis shows the cumulative share of residents from lowest to highest greenspace exposure.


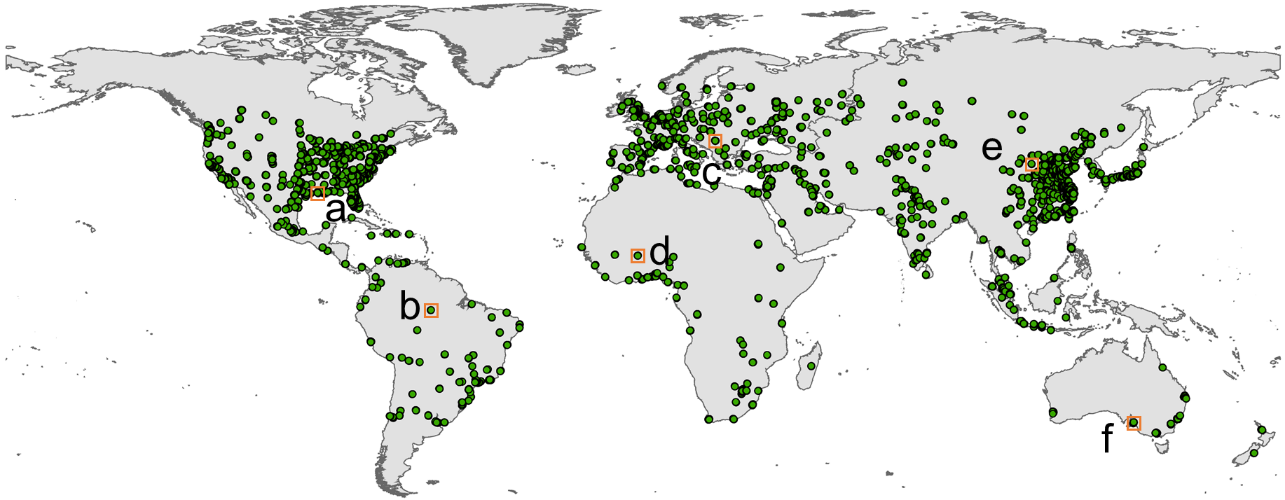


**Supplementary Fig. 15. Geographic distribution of validation samples (greenspace and non-greenspace) across 1028 cities globally. a-e**, Locations of zoomed-in subsets of Figure S2.


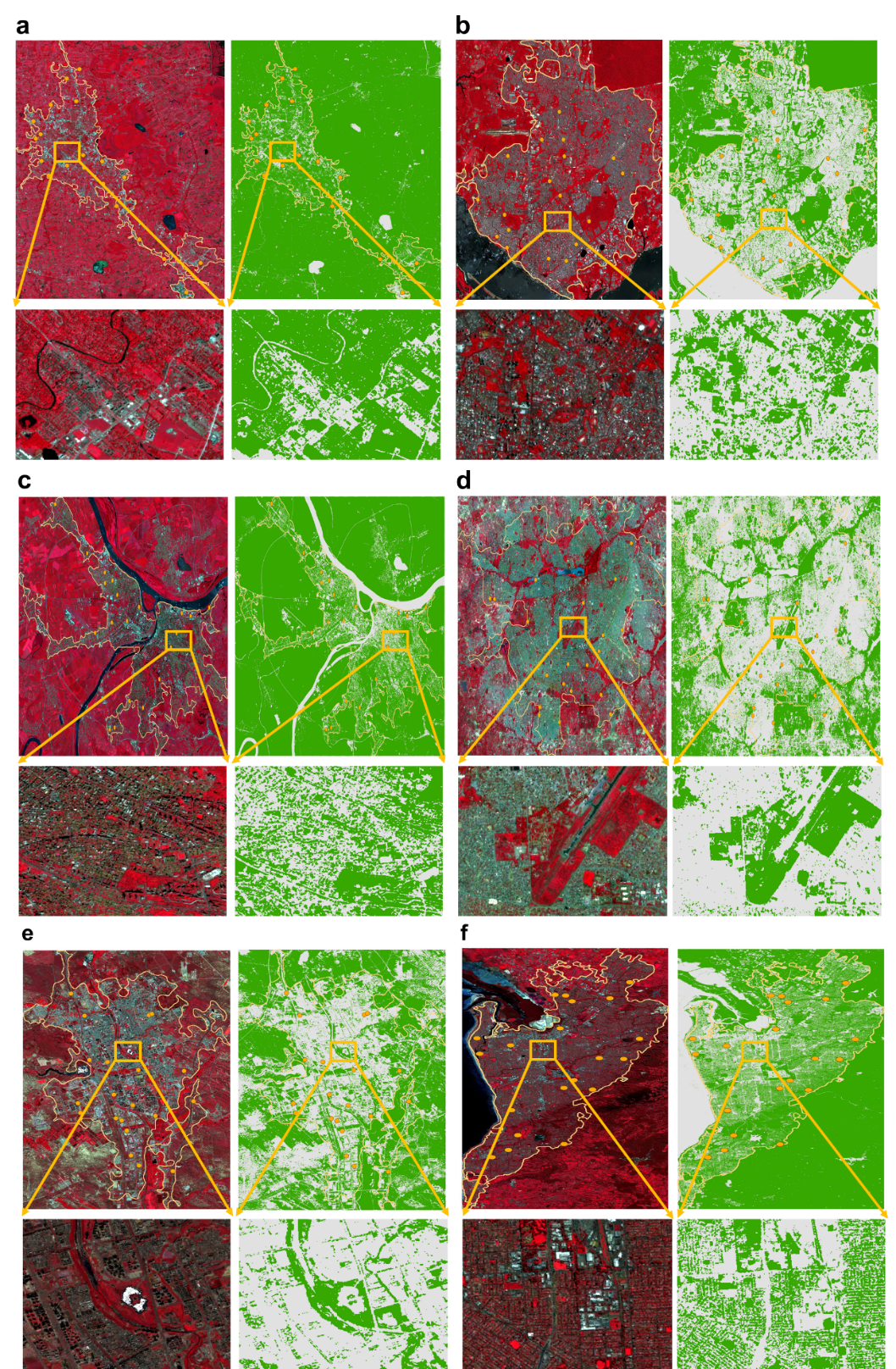


**Supplementary Fig. 16. Comparison of Sentinel-2 imagery and the associated greenspace and non-greenspace classification in zoomed-in cities (a-e) across the globe.** The green color represents the greenspace coverage and gray color represents non-greenspace coverage. The yellow dots are validation samples derived from visual inspections.

**
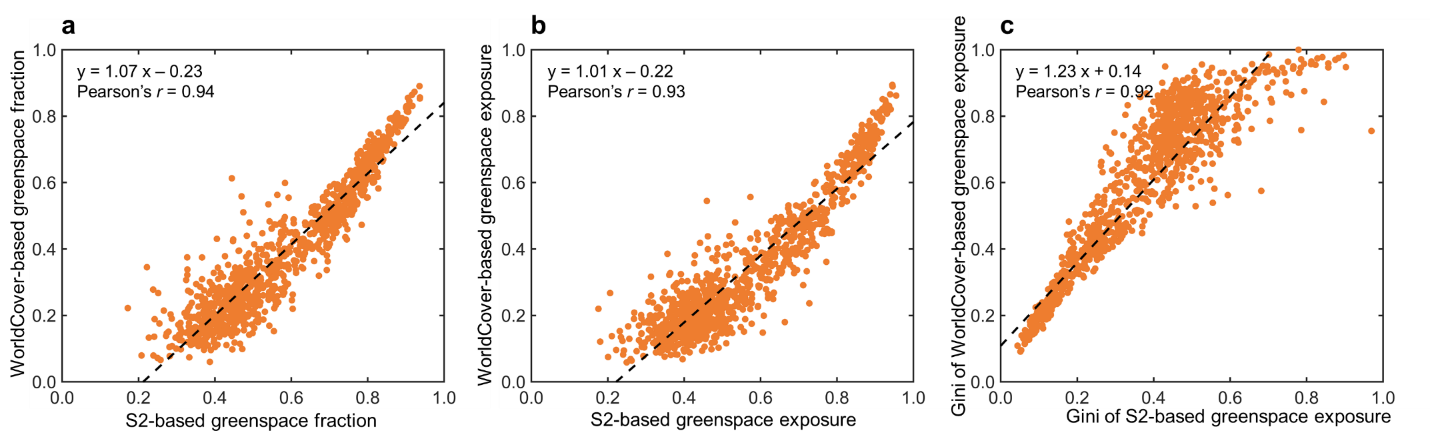
**

**Supplementary Fig. 17. Comparison of greenspace fraction (a), greenspace exposure estimates (b), and Gini index of greenspace exposure inequality (c) derived from Sentinel-2 imagery (x-axis) and WorldCover product (y-axis) across global 1028 cities.** Linear regression was used to measure their correlation with Pearson’s r coefficient.

**
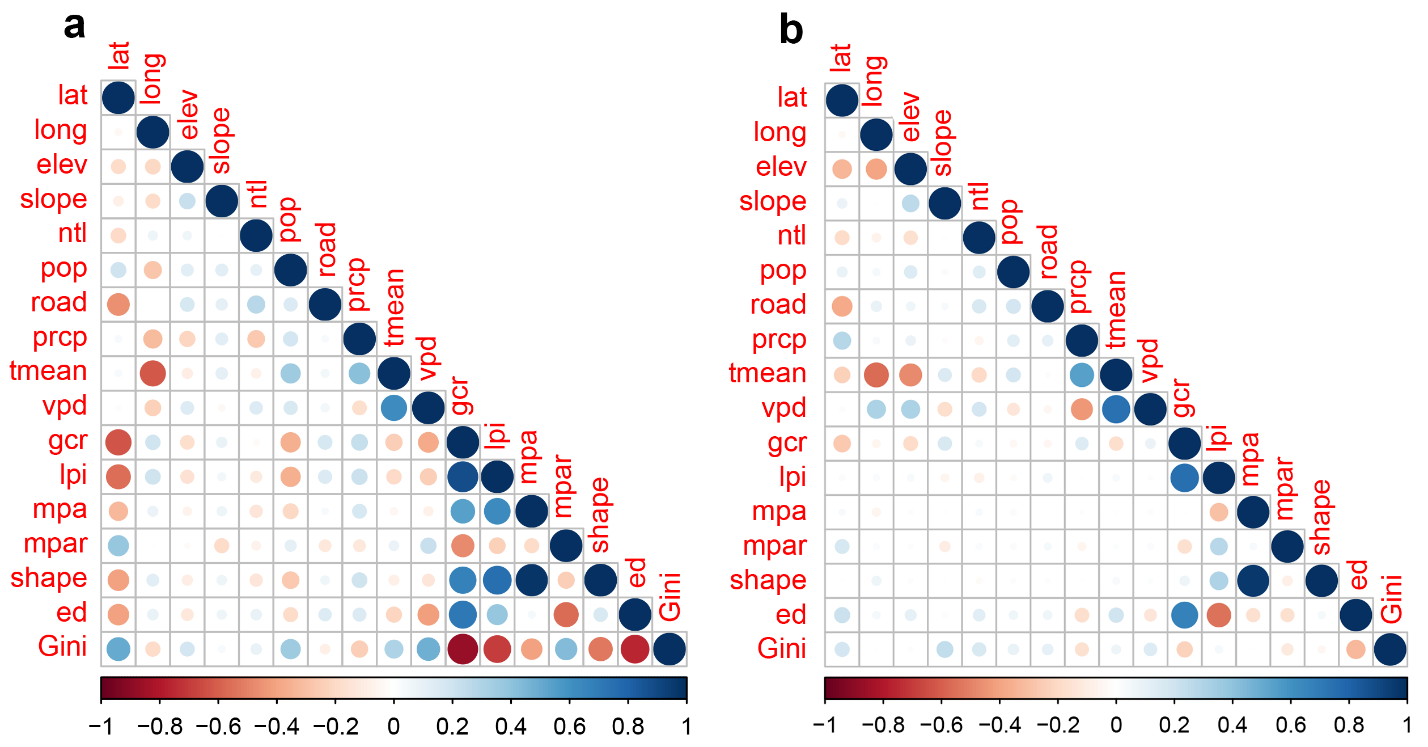
**

**Supplementary Fig. 18. Correlation matrix between explanatory variables and urban greenspace exposure inequality (measured by Gini of greenspace exposure).** a, Pearson’s correlation matrix and b, Partial correlation matrix.

**
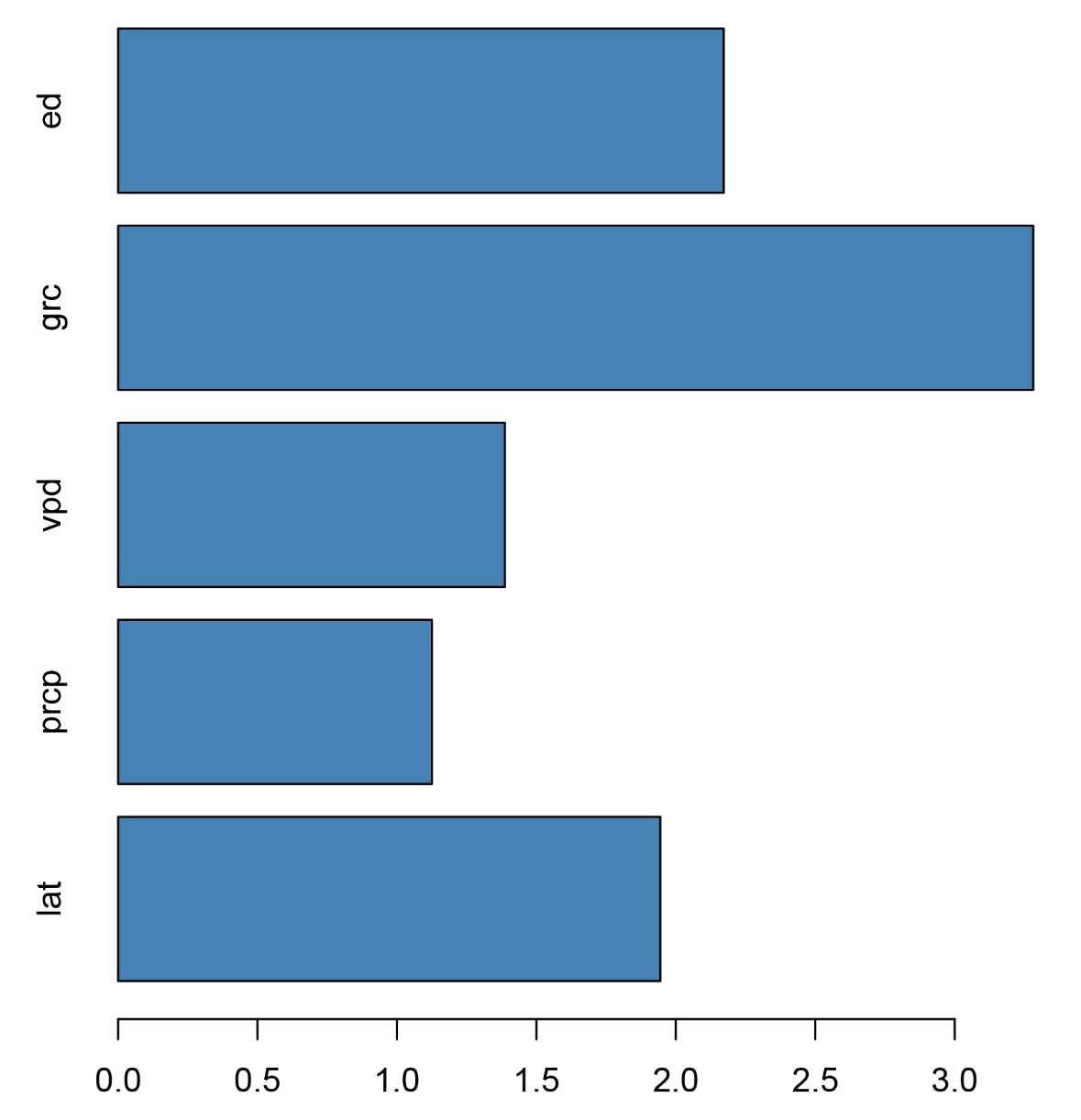
**

**Supplementary Fig. 19. Bar summary of Variance Inflation Factors (VIF) for five inclusive variables in the multiple linear regression model.**

**
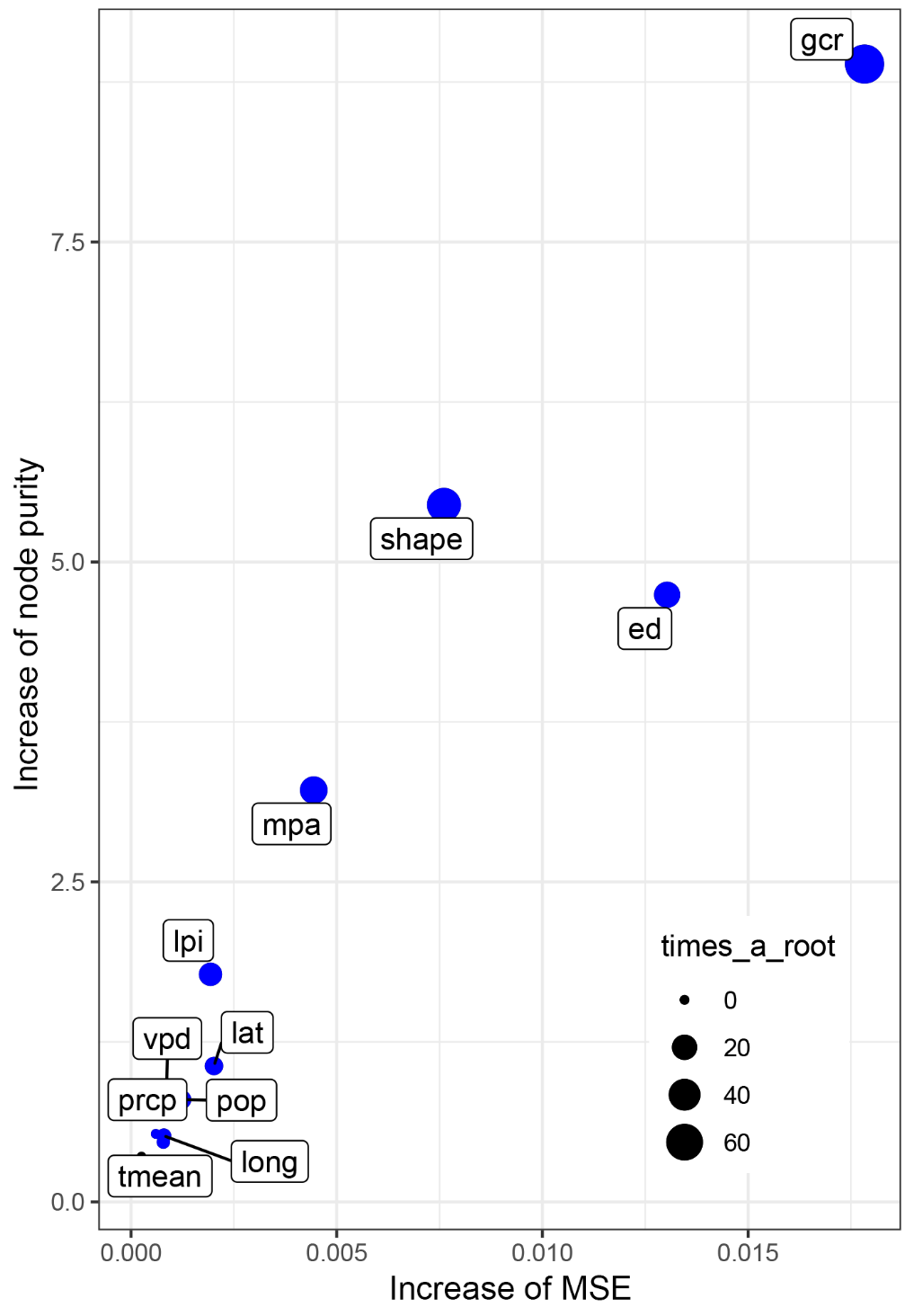
**

**Supplementary Fig. 20. Variable importance from the random forest model, measured by x-axis (the increase of mean square error after excluding specific variable), and y-axis (the increase of node purity after including specific variable).** The dot size represents the number of trees in which the specific variable is used for splitting the root node.


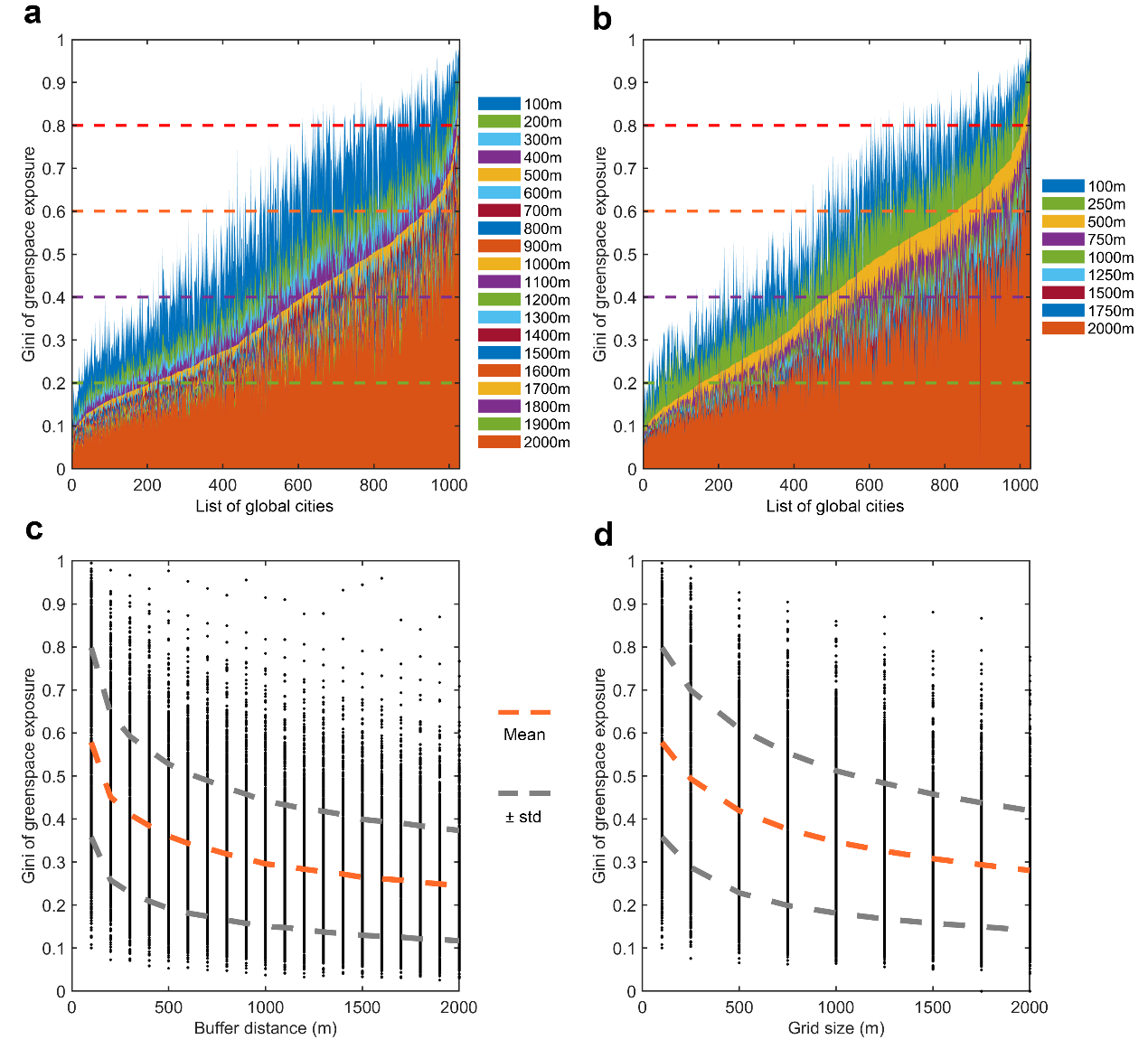


**Supplementary Fig. 21. Sensitivity of buffer distance and grid size to the assessment of greenspace exposure inequality, as measured by Gini index for global urban areas. a,** Gini of greenspace exposure estimated using buffer zones from 100 m to 2000 m with an interval of 100 m. The list of 1028 urban areas are ordered by the Gini of Greenspace exposure measured using the 500-m buffer zone. **b,** Gini of greenspace exposure estimated using grid sizes from 100m, 250 m to 2000 m with an interval of 250 m, without considering buffer zones. The list of 1028 urban areas are ordered by the Gini of Greenspace exposure measured using the 500-m grid size. **c,** The variation of Gini of greenspace exposure to buffer distance. **d,** The variation of Gini of greenspace exposure to grid size.

**Supplementary tables**

**Supplementary Table 1.** Correlation coefficients between explanatory variables and the Gini of greenspace exposure. The partial correlation coefficient is derived by controlling all the other variables.

| Category | Variable | Correlation | Partial correlation |
| --- | --- | --- | --- |
| Geographic | lat | **0.51^***^** | **0.19^***^** |
|  | long | -0.19**^***^** | 0.01 |
| Topographic | elev | 0.18**^***^** | 0.01 |
|  | slope | 0.04 | 0.24**^***^** |
| Socio-economic | ntl | 0.10^*^ | 0.16**^***^** |
|  | pop | 0.36**^***^** | 0.10^*^ |
|  | road | -0.08^*^ | 0.10**^**^** |
| Climatic | prcp | **-0.25^***^** | **-0.16^***^** |
|  | tmean | 0.31*** | 0.07 |
|  | vpd | **0.49^***^** | **0.15^***^** |
| Landscape | grc | **-0.87^***^** | **-0.22^***^** |
|  | lpi | -0.70**^***^** | 0.05**^***^** |
|  | mpa | -0.40**^***^** | 0.002 |
|  | mpar | 0.44**^***^** | -0.12**^*^** |
|  | shape | -0.53**^***^** | -0.04 |
|  | ed | **-0.76^***^** | **-0.32^***^** |

**Note:** ^***^ p-value < 0.0001, ^**^ p-value < 0.001, ^*^ p-value < 0.01

**Supplementary Table 2.** Accuracy validation of Sentinel-2 based greenspace mapping across global urban areas.

| Reference  WorldCover | Greenspace | Non-greenspace |
| --- | --- | --- |
| Greenspace | 11,070 | 542 |
| Non-greenspace | 688 | 8226 |

**Note:** Overall accuracy: 0.94, Precision: 0.94, Recall: 0.95, and F1-score: 0.95.
